# The Tight-interlocked Rhythm Section: Production and Perception of Synchronisation in Jazz Trio Performance

**DOI:** 10.1080/09298215.2017.1355394

**Published:** 2017-07-31

**Authors:** Alex Hofmann, Brian C. Wesolowski, Werner Goebl

**Affiliations:** ^a^ University of Music and Performing Arts Vienna, Austria.; ^b^ Austrian Research Institute for Artificial Intelligence (OFAI), Austria.; ^c^ The University of Georgia, USA.

**Keywords:** synchronisation, jazz ensemble, timing, drums, bass, saxophone

## Abstract

This study investigates the production and perception of timing, synchronisation and dynamics in jazz trio performances. In a production experiment, six trio combinations of one saxophonist, two bassists, and three drummers were recorded while they performed three popular jazz songs. Onset timing and dynamics of each performer were extracted and analysed. Results showed that the tempo was significantly influenced by the timing of the drummers and all performers showed higher temporal precision on the backbeats. The drummers demonstrated individual swing-ratios, accentuations of beats and intrapersonal asynchronies between simultaneous hi-hat and ride cymbal onsets, which resulted in a hi-hat played 2–26 ms ahead of the pulse of the music. In a subsequent perception test, participants (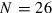) rated 12 excerpts of the jazz recordings. They selected their preferred version from a pool of stimuli containing the original version, but also manipulations with artificially increased or reduced asynchronies. Stimuli with reduced asynchronies smaller than 19 ms were preferred by the listeners over the original or the fully quantised timing. This suggests that listeners endorse a ‘tight-interlocked’ jazz rhythm section, with asynchronies smaller than the perceptual threshold (temporal masking), but with natural timing variabilities that makes it distinguishable from a computer-generated playback.

## 1. Introduction

Audiences are fascinated by how professional jazz musicians form a partnership on stage to create music together. The creativity and a mutual understanding of each performer’s skills contribute to the ensemble performance, which, through its improvisatory nature, is different each time, even for familiar repertoire (Burland & Pitts, 2010). Jazz musicians have a highly trained auditory sensitivity for timing and melodic contours, which allows them to reshape a song on the fly and interpret it in their unique way while maintaining the groove of the music (Tervaniemi, Janhunen, Kruck, Putkinen, & Huotilainen, 2016).

The main motivation of this study is to observe interaction in a jazz ensemble, its influence on the timing and the dynamics of the music, and the listeners’ perception of the latter. Jazz performances are often characterised by their unique groove. Besides the active scientific discussion on possible properties in the music that might contribute to the perception of groove, there is a common agreement that groove is a quality of music that makes listeners want to move or dance to the music (Frühauf, Kopiez, & Platz, 2013; Janata, Tomic, & Haberman, 2012; Kilchenmann & Senn, 2015; Madison, 2006).

When musicians play together, interpersonal coordination in the ensemble depends on (a) the ability to predict the upcoming actions of the partner, and (b) the ability to adapt to the occurring actions of the partner (Konvalinka, Vuust, Roepstorff, & Frith, 2010). When musicians synchronise with each other, they can either use auditory or visual cues to inform their predictions (Bishop & Goebl, 2015). Except for the style of ballads, jazz performances are typically played in a steady time feel with a constant pulse (Wesolowski, 2013, 2016). Here, all ensemble members have to synchronise on the microtiming level and follow the same tempo.

Nevertheless, timing profiles of jazz ensembles are far from perfect. An aim of this study is to measure how each player affects the timing and the tempo of the entire ensemble.Asynchronies between tone onsets occur during every performance and the performers have to adapt, so that the synchronisation error does not accumulate over time (Butterfield, 2010; Goebl & Palmer, 2009; Konvalinka et al., 2010). The occurrence of asynchronies in music ensembles can be explained by the natural variability in either the timekeeper or the motor system of each player in the ensemble (Goebl & Palmer, 2009; Hofmann & Goebl, 2014; Repp, 2005c). Deviations in the tempo and asynchronies between the instruments in a jazz ensemble raise the question of whether asynchronies contribute to the perception of groove or not.

Keil (1987) and Prögler (1995) measured vertical timing deviations in a jazz rhythm section, more specifically between the bassist’s note onsets and the drummer’s ride cymbal taps. They reported that these ensemble asynchronies create a listening experience of tension and release on top of the regular underlying pulse of the music and termed these ‘Participatory Discrepancies’ (PDs). PDs were said to contribute to the experience of groove in music. However, later studies which used systematic manipulations of (computer generated) jazz beats were not able to support the PD theory, failing to find a positive influence of PDs on the perception of groove (Butterfield, 2010; Frühauf et al., 2013; Janata et al., 2012; Madison, 2006; Madison & Sioros, 2014). Kilchenmann and Senn (2015) measured spontaneous periodic movements of non-expert and expert music listeners while they listened to manipulated jazz beats with more or less asynchronies. Entrainment in the form of stronger head movements was only found for the group of expert listeners, when they listened to reduced PDs (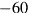
% smaller asynchrony than recorded with human musicians). With an associated questionnaire they found that for larger PDs, both groups of listeners reported to be irritated and stimuli with reduced asynchrony (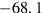
%) got better ratings on entrainment (Senn, Kilchenmann, von Georgi, & Bullerjahn, 2016). In this study, the sound material contained only a drummer and a bassist playing together. Listening to the rhythm section, without any melody instrument may result in an unnatural listening focus on the accompaniment in contrast to listening to jazz music which mostly features soloists.

When jazz soloists are on tour, it is common practice for them to be accompanied by a local rhythm section they have never played with before (Burland & Pitts, 2010). Particularly in jazz music, where timing and groove is an important aspect of the music, the timing of one player in the rhythm section might influence the quality of the entire ensemble (Goolsby, 1997; Wesolowski, 2016, 2015). In this study, we are particularly interested in how listeners evaluate jazz recordings from different ensembles. To analyse effects of a particular player, we recorded combinations of a jazz trio with one saxophonist, two bassists and three drummers and presented these recordings to listeners with different experiences in music making and dancing.

Since the 1980s, dance music producers often use studio technology that allows them to align the instrument sounds (preferably drums and bass) with computer-controlled timing precision (quantisation; Butler, 2006). Such perfectly synchronised music is also called *quantised* or *dead-pan*. Electronic dance music (EDM), which is commonly played during dance events, is quantised. In EDM, other musical properties like syncopation or timbre variations were found to have a positive effect on the motivation of listeners to move or dance to the music (Wesolowski & Hofmann, 2016; Witek, Clarke, Wallentin, Kringelbach, & Vuust, 2014). Timbre variations and syncopation can also be found in jazz performances, and today listeners are used to dancing to quantised music. We hypothesise that this might have changed the listening habits over the last decades and listeners may nowadays endorse quantised beats also for jazz ensembles. However, there might be an exception for jazz soloists. Soloists use expressive timing with asynchronies in a range of 50–80 ms to the underlaying beat to emphasise and contour their improvisations (Benadon, 2009). Nevertheless, such expressive playing techniques can only be applied if the rhythm section generates an isochronous *pulse* (Doffman, 2009).

In jazz music, specific attention is given to the relative timing of the eighth notes. The *swing-ratio* has been defined as the timing proportions of consecutively played eighth notes (Friberg & Sundström, 2002). In a medium tempo swing style, the second eighth note is usually delayed but played with a shorter duration to maintain an even quarter beat pulse (see Wesolowski, 2013 for an overview). Although jazz drummers play the eighth note swing pattern on the ride cymbal, the pedal hi-hat plays a constant half time pulse on the beats 2 and 4 of each bar, which is called the *backbeat* (Butterfield, 2006). When a band leader is counting-in to give the tempo to the ensemble, he emphasises the backbeats.^1^ Parsons and Cholakis (1995) looked at drum patterns of 15 professional jazz drummers and found that the backbeats were dynamically emphasised compared to the downbeats (1 and 3). They reported that some drummers used alternated beat durations for downbeats and backbeats which causes a variation in the regularity of quarter notes, but still keeps a consistent half-note tempo. An aim of this study is to differentiate between a general playing style when performing jazz and a personal expressive playing style, by comparing the timing and dynamics of the recorded players.

Drummers can play multiple instruments at the same time. A drummer can control the intrapersonal synchronisation of the sounds by the timing used to trigger the drum instruments with both drum sticks and the pedals operating the bass drum (right foot) and the hi-hat (left foot). Intrapersonal asynchronies in drum performances have been studied from different perspectives, which ranged from the synchronisation error of inter limb synchronisation (Fujii et al., 2011) to the effect of systematic delays of certain drum instruments on the perception of groove (Butterfield, 2006; Frühauf et al., 2013). Taking the intrapersonal synchronisation of the drummers into account, the asynchronies between the ride cymbal and the hi-hat might be of particular interest to characterise the playing style of a drummer and its influence on the synchronisation of the entire jazz ensemble.

A goal of this study is to investigate the influence of varied personnel in the jazz rhythm section on the timing and the dynamics in the music. Therefore, we recorded six professional jazz musicians in different ensemble combinations to (a) compare the overall timing of the ensembles, (b) the individual differences in timing and dynamics of the performers, and (c) the asynchronies that occur in the ensembles. In a subsequent perception experiment, we aim to evaluate the properties of the music by analysing listeners’ ratings given for these recordings with original and manipulated ensemble asynchronies.

## 2. Production experiment

### 2.1. Methods

#### 2.1.1. Materials

We selected three popular jazz song forms: (a) based on the song ‘Have you met Miss Jones’ by R. Rodgers and L. Hart [medium swing; 168 bpm], (b) a 12-bar blues form in C for Alto Sax [fast swing, 208 bpm] and (c) based on the song ‘On Green Dolphin Street’ by B. Kaper and N. Washington [Latin/Swing; 168 bpm]. In jazz music, several reharmonizations of the same song do exist and are equally often played (Shanahan & Broze, 2012). To ensure that all participants play the same version of the song, we provided chord charts, although all participants reported to be familiar with the song forms and that they have performed them multiple times before.

#### 2.1.2. Participants

Six professional, American jazz musicians from the area of Atlanta (Georgia, USA) took part in this study (2 bassists, 3 drummers, 1 saxophonist). The saxophonist had more than 35 years of professional playing experience. Drummer D1 had 7 years of professional playing experience, drummer D2 had 25 years playing experience, and drummer D3 played professionally for about 8 years. Bassist B1 reported 5 years of professional playing experience, and bassist B2 had more than 35 years of playing experience.

#### 2.1.3. Experimental setup

The recordings were made in a rehearsal studio in the *Hugh Hodgson School of Music* at the *University of Georgia*. Each trio-ensemble played simultaneously in the same room, which allowed uninterrupted eye contact during performance. To capture clean signals for each participant, we used electronic instruments for the drums and the bass. The set-up allowed them to hear each other through professional studio headphones (MDR-7506, by Sony). An Electronic V-Drumkit (TDKS-V Compact TD11 Sound Module Setting: SwingJazzKit, by Roland) provided both the sound of a jazz drumkit and MIDI data of the played drum notes. An electric double bass (NXT-4 String Electric Double Bass, by NS Design) was used to capture the clean sound of the bass. Each of the three players used headphones in order to hear the sound of the drummer and the bassist. A sub-mixer allowed them to adjust their volumes individually. For the alto-saxophone, a clip microphone (d:vote 4099, by DPA) on the bell of the instrument was used to capture the direct sound signal.

Additionally, we attached accelerometers to each of the drum sticks to capture the movements of the sticks. Accelero-metres on the right-hand index finger and the middle finger of the bassists tracked their plucking gestures. The finger movements of the saxophonist were captured with eight accelerometers, together with the tongue articulation using a strain gauge sensor on the reed (Hofmann & Goebl, 2014).

All the MIDI, audio and sensor signals were recorded simultaneously using a high-quality multi-channel combined audio/MIDI studio interface (Scarlett 18i20, Octopre MkII, by Focusrite; 48 kHz, 24 Bit, A/D conversion) and the Ableton Live 9.1 software on a MacBook Air computer (OSX 10.10, by Apple, Inc.). Using one input device for all data streams, timestamps for incoming information were processed by the same hardware clock of this interface. These timestamps are directly saved as double precision numbers in the Ableton Live software project file (DeSantis et al., 2016, p. 661).

#### 2.1.4. Procedure

The musicians were grouped into six possible combinations of a jazz trio ensemble, each containing a bassist, a drummer and the saxophonist. Each ensemble improvised twice over the form of the three jazz songs. Previous research indicates that the incorporation of isochronic tones in the context of sensorimotor processing synchronisation studies, such as those stemming from a metronome, affect accent production (Billon & Semjen, 1995; Repp, 2005b), synchrony (Repp, 2005a), phase correction (Repp, 2008), and self-generated interval subdivision (Repp, 2010). Therefore, in order to glean authentic timing data, a metronome was not used throughout the performances. The tempo was introduced in the beginning of each trial with a digital metronome and turned off when the band started to play, similar to the way a band leader introduces the tempo before the ensemble starts to play under normal performance conditions. Two trials per song form were recorded. Each trial had the duration of 2 choruses of the form. The total corpus of data contains 45 min of recordings.

#### 2.1.5. Data analysis

To gain precise timing information about each performer the physical note-onsets and note-offsets were extracted from the captured data and manually checked for every instrument and every recorded trial.

For the drums, we checked the captured MIDI data from the triggers of the drum-kit, by comparing the MIDI onsets with the recorded sound of the TD11 sound module and the data from the accelerometers on the drum sticks. We noticed a consistent delay of the sound by 2 ms after a peak in the accelerometer data and 5 ms delay of the following MIDI event. To compensate for this constant MIDI delay, we shifted all drum MIDI notes by these 5 ms to be aligned with the sound.

To extract timing information from the recorded audio signals of the bass-pickup and the saxophone, the Ableton software was used for annotation (DeSantis et al., 2016, Convert Melody to New MIDI Track, p. 174). The algorithm searches for transients in the sound (DeSantis et al., 2016, p. 141) and transcribes note onsets at the very beginning of a change in the sound. The annotations were manually checked by comparing the detected events with the audio signals and the sensor signals of the accelerometers on the fingers of the players. For the checked onsets, the time difference between the annotated onset and the actual tone onset was less than 1 ms. False onset detections occurred in one trial when the recorded signal was interfered for 5 s and when multiple strings of the bass were producing sound. Such rare false positives were manually deleted from the annotation, but no manual correction of onset positions was applied nor were missing onsets (false negatives) manually added, to avoid a bias through this post-processing of the timing data.

The timing values of the note events were directly imported into R-statistics for further statistical analysis.^2^ The entire dataset comprised more than 38,000 captured note events stored in one concatenated list, containing all recorded trials which are used for the following timing analysis.

### 2.2. Results

#### 2.2.1. Overall timing

We calculated the mean signed timing error based on inter-onset intervals (IOI), the time span between two consecutive quarter note onsets played by the bass (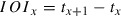). The mean signed timing error is a measure for the relative deviation from the introduced tempo and is calculated based on the IOI sequence: 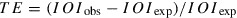.

**Figure 1.**
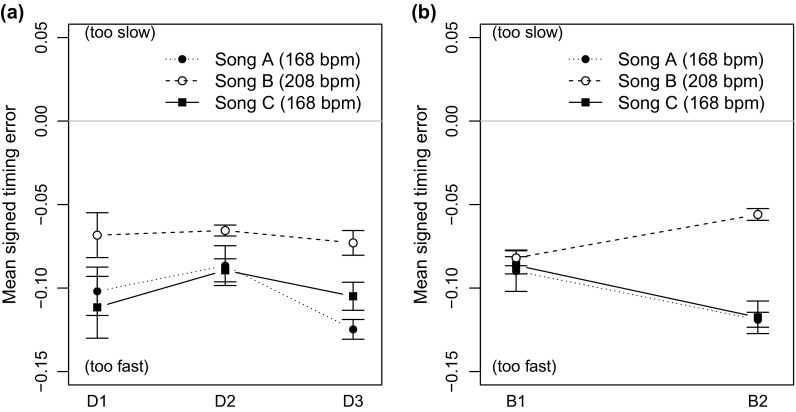
Timing Error for the three songs, grouped by the drummers (a) and the bassists (b) playing in the ensemble. The error bars show the standard error of the mean.

The overall negative mean signed timing error for all recorded trials of the different ensemble combinations (between 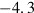 and 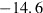
%) shows that all trios played too fast. A three-way analysis of variance (ANOVA) on the timing error by drummer, bassist and song as factors showed a significant main effect of the performed song [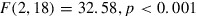], and the drummer [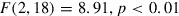, see Figure 1(a)], but no main effect for the bassists. Although all three songs were played with a faster tempo than introduced, in particular the slower songs (A) and (C) showed more increase in tempo (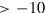
%) than the faster Blues (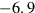
%). The main effect for the drummer indicates a dominant influence of the drummer on the overall timing of the ensembles. A significant interaction of bassist and phrase [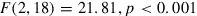, see Figure 1(b)] indicates individual timing profiles for the bassists with the three songs. Here, ensembles including B2 showed less tempo acceleration for song (B) than the remaining ensembles. Finally, the three-way interaction of drummer, bassist and song [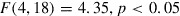] can be interpreted as an indicator how each ensemble developed its individual timing for each of the performed songs.

To investigate the temporal precision of the quarter note onsets in the rhythm section (walking bass, drum beat sounds) we calculated the coefficient of variation (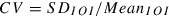) from the sequence of tone onsets played on quarter note beats by the bassists and the drummers. CV values are a measure for the regularity of the note events, with values close to zero indicating high regularity and larger values showing variability in the distribution of the onsets. We used a mean onset time when multiple instruments (e.g. bass drum, ride cymbal, hi-hat and bass note onset) were played simultaneously. (An analysis of intrapersonal timing of the drummers can be found in Section 2.2.2 in this paper.) From the sequence of bass and drum quarter note onsets, a coefficient of variation of CV = 0.074 indicates high regularity in the note events for the rhythm section players. Timing precision values between CV = 0.03–0.08 have been reported for professional pianists performing an isochronous melody depending on the playing tempo (Goebl & Palmer, 2008).

**Figure 2.**
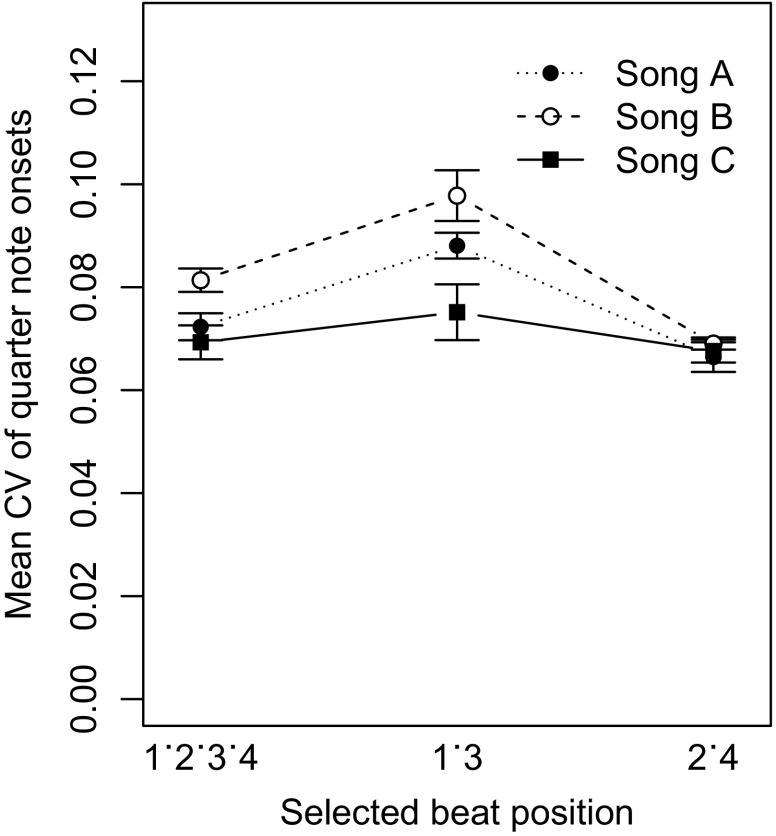
Coefficient of variation (CV) of quarter note onsets played by the rhythm section players grouped by the three songs. Onset variability was higher for beats 1 and 3 than for the backbeats (2 and 4). The error bars show the standard error of the mean.

A three-way ANOVA on the CV by drummer, bassist and song as factors showed a significant main effect of the song which was performed [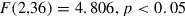] but no effect of the players, or any interactions. However, an analysis on the next higher metrical level of half notes showed a significantly higher precision for onsets played on the beats 2 and 4 (CV = 0.068) than for the beats 1 and 3 (CV = 0.087) [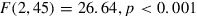]. Figure 2 shows that this was the case for all three songs, but with a stronger effect for the songs in swing style (A) and (B) than for the Latin jazz song (C).

#### 2.2.2. Individual differences in the drum performances

Musical expression in jazz rhythms has been characterised by three main properties: (a) by the timing relation of consecutive eighth notes also reported as the swing ratio, (b) by the placing of the onsets in relation to the metrum of the music, and (c) by the dynamics of the note events (Wesolowski, 2013). In the following section, we will examine the properties of the drum beats performed by the three different players.

##### 2.2.2.1. Swing ratios of drummers

To analyse the swing-ratios used by the different drummers, the dataset was reduced to the two songs which were played in swing style (A and B). From the MIDI data captured from the full drum-set, only the ride cymbal onsets were used for this analysis. We calculated the swing ratio of subsequent eighth notes, by dividing the onset-to-onset duration of the first eighth note by the onset-to-onset duration of the second eighth note, following the procedure given in Friberg and Sundström (2002).

**Figure 3.**
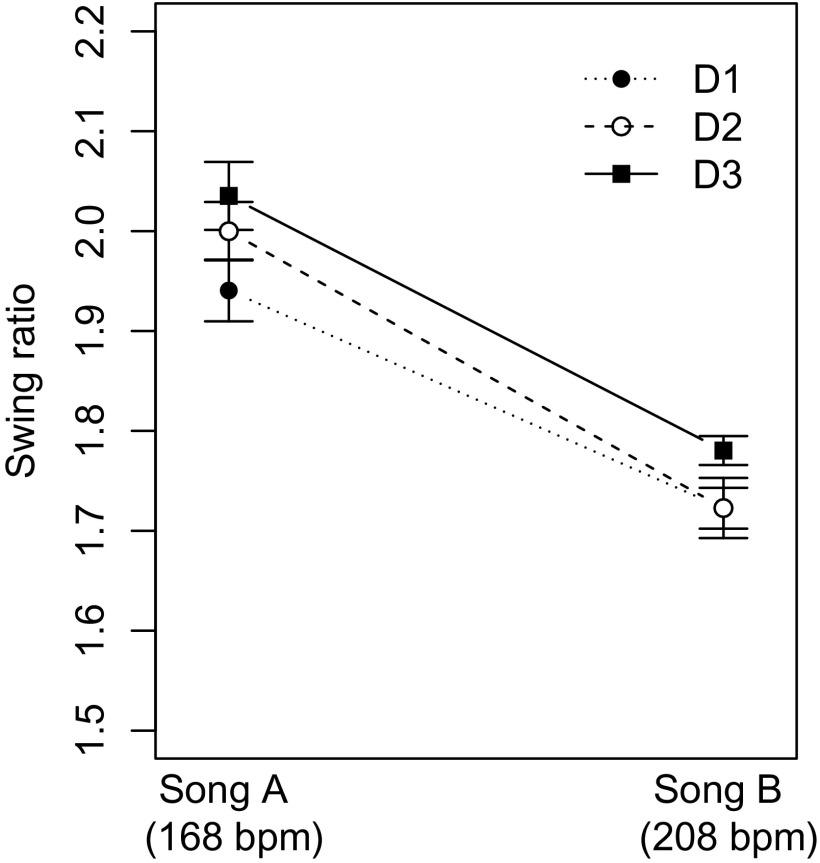
Swing ratios measured for all three drummers’ cymbal taps, grouped by the two different swing songs (A and B) played with different tempi. The error bars show the standard error of the mean.

A three-way ANOVA on the measured swing ratios by drummer, bassist and song as factors confirmed a significant main effect of the two songs (with different tempi) [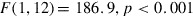], as well as a significant main effect of the drummer [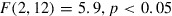]. Figure 3 shows that the swing ratios were significantly larger for the medium tempo swing (168 bpm) than for the Blues (208 bpm). Moreover, it is visible in Figure 3 that D3 used larger swing ratios for both songs than the other two drummers. The observed swing ratios of all drummers are in the range of the values reported for professional drummers by Friberg and Sundström (2002).

##### 2.2.2.2. Dynamics of drummers

**Figure 4.**
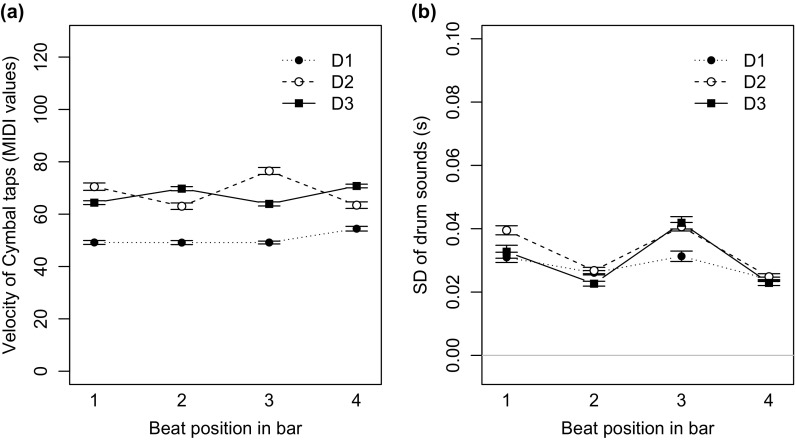
(a) Velocities of cymbal taps on the quarter beats showed individual accentuation patterns. (b) Standard deviation of drum onset times falling on the same quarter beat position were smaller for the backbeats 2 and 4. The error bars show the standard error of the mean.

Among the velocities of the ride cymbal taps falling on the quarter beat notes, we observed individual patterns for all three drummers. Figure 4(a) shows that D3 emphasises beat 2 and 4. This accentuation pattern has been reported to be the most popular (Butterfield, 2006). However, D2 shows an inverted accent pattern, where the beats 1 and 3 are emphasised. Moreover, D1 is using balanced dynamics in the ride cymbal taps on all four beats. This indicates that additionally to the swing ratio, accents of the ride cymbal may be an important characteristic of personal style in jazz performance and may not necessarily follow a predefined pattern.

##### 2.2.2.3. Asynchronies within the drums

From the MIDI notes of the drum beats, we calculated the time difference between cymbal taps (CY) falling on the same beats with the pedal hi-hat (CY–HH = 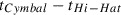). A positive cymbal to hi-hat asynchrony value (CY–HH) indicates that the cymbal was played after the hi-hat.

Overall all three drummers were delaying their cymbal pattern in relation to their hi-hat. Drummer D1 showed the largest mean signed CY–HH asynchrony with +27.9 ms, followed by D3 (CY–HH = +13 ms) and D2 (CY–HH = +8.6 ms). Taking into account that professional jazz drummers are able to control their timing in the range of milliseconds (Honing & De Haas, 2008), this shows that all three drummers were using different degrees of the asynchrony between their cymbal and hi-hat sounds to emphasise their backbeats (2 and 4).

Another measure for asynchrony is the standard deviation (SD) of drum onsets played on the same beat. A larger SD is an indicator for larger (vertical) microtiming deviations. Figure 4(b) shows that all three drummers were using smaller asynchronies on the backbeats (2 and 4) than on beats 1 & 3. This underpins the importance of the backbeats in jazz rhythms.

#### 2.2.3. Ensemble asynchronies from the perspective of PDs

##### 2.2.3.1. Asynchronies within the ensemble

The synchronisation of jazz ensemble members has been a popular study object in the last decades to investigate the occurrence of groove in music (e.g. Butterfield, 2010; Keil, 1987; Prögler, 1995). Most of these studies measured timing deviations between the drummer’s ride cymbal taps and the bassist’s note onsets. To generate comparable data, we calculated the time differences between ride cymbal taps (CY) and bass onsets (BS) falling on the same quarter note beats by CY–BS 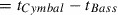. With this measure, positive values indicate that the bass onsets were played earlier than the cymbal hits and negative values denote a bass playing in a laid-back fashion.

**Figure 5.**
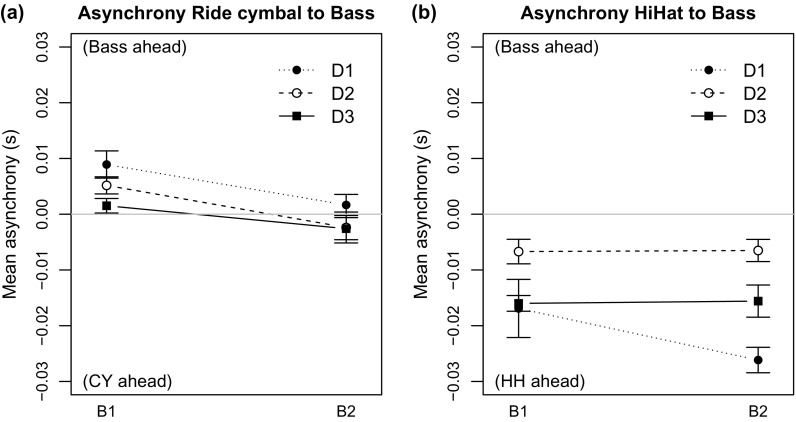
Asynchronies between the bass note onsets and the (a) cymbal taps indicate that bassists are playing on the top of the beat, whereas asynchronies to the (b) hi-hat indicate the bassists to play laid-back. The error bars show the standard error of the mean.

Figure 5(a) shows that all ensemble combinations played in almost perfect synchrony, averaging to a mean signed asynchrony of CY–BS = +2.1 ms. A three-way ANOVA was conducted, to compare the effect of the ensemble members and the different songs on the CY–BS asynchrony. We found a significant main effect of bassist [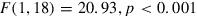] as well as a significant effect of drummer [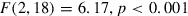], but no effect for songs was found. These results suggest that how players interlink is not dependent on the song played but on the combination of personal playing styles. Moreover, the two significant interactions for song-bassist [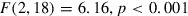] and song-drummer [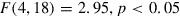] show the individual differences between performers’ timing profiles. Figure 5(a) shows that the mean asynchrony was slightly larger for B1 (CY–BS = +5.2 ms) than for B2 (CY–BS = 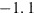 ms). The effects of the three drummers were within the same range of asynchronies (CY–BS: D1 = +5.3 ms, D2 = +1.4 ms, D3 = 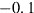 ms). From this analysis, we conclude that the ensembles played in almost perfect synchrony and no systematic timing effects of bass or drums occurred.

However, taking the observed intrapersonal asynchronies of the drummers into account, we were interested in the asynchronies between the backbeat pattern of the hi-hat (HH) and the bass onsets. Doing the same calculations for the hi-hat onsets (HH–BS=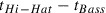) showed that all three bassists played in a laid-back fashion (7–26 ms) in relation to the hi-hat (see Figure 5(b)). The same three-way ANOVA for the HH–BS asynchrony revealed a significant main effect of drummer [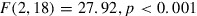], but no effect of bassist was found [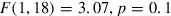].

Figure 5(b) shows that both bassists synchronised better with D2 than with D1 or D3 (HH–BS: D1 = 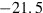 ms, D2 = 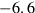 ms, D3 = 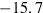 ms). The smallest intrapersonal asynchronies were observed for D2, which may enable smaller interpersonal ensemble asynchronies. Bassists seemed to align their playing to the CY, while drummers can manipulate asynchronies between the individual drum instruments to create expressive effects.

##### 2.2.3.2. Asynchronies within the ensemble using a mean pulse as reference

In music, and especially in groove-based music, listeners and performers are able to anticipate upcoming beats, based on tempo and timing expectations established through previous events (Huron, 2006, p. 184). Dixon, Goebl, and Cambouropoulos (2006) argued that the tempo perceived by musically trained listeners might be closer to a smoothed rendition of the measured timing data than the raw data and provided a perceptual timing model taking vertical and horizontal smoothing into account. Based on this model, we computed an overall *pulse* for each performance.

The computation of the overall *pulse* was as follows: the mean onset times for all notes (from the entire ensemble) falling on the same quarter-note beat (vertical) were calculated. From this sequence of mean ensemble onset times 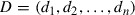, a smoothed *pulse* sequence 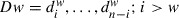 was computed. Each onset time 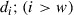 in the sequence was smoothed by a moving average (WMA) spanning a window of 4 quarter notes on either side (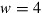), weighted by a Gaussian curve [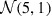]:(1)
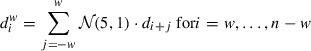



To calculate the asynchronies for a specific instrument (X) from the ensemble, we subtracted the onset times from the *pulse* (Pulse–X 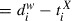). A positive value indicates an onset played before the *pulse*, a negative value indicates an onset played after the *pulse*.

**Figure 6.**
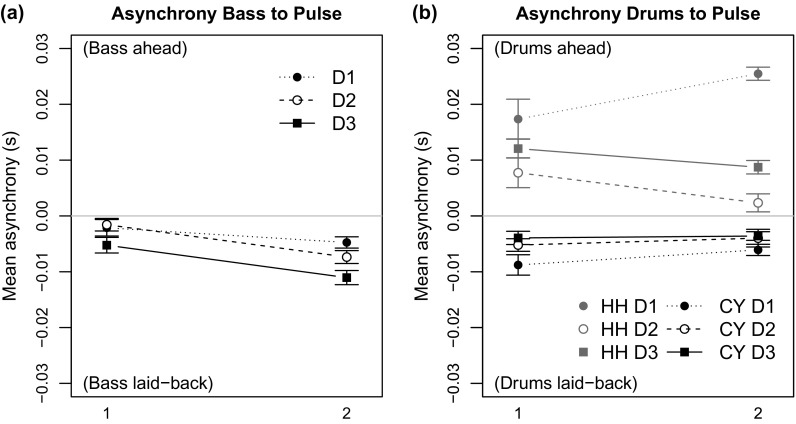
(a) Asynchronies between the *pulse* and the bass note onsets indicate that the bassists played in a laid-back fashion. (b) Cymbal taps (black) were played slightly after the pulse, and the hi-hat (grey) is on top of the *pulse*. The error bars show the standard error of the mean.

Figure 6(a) depicts the mean signed asynchronies of the bassists to the *pulse*. It shows that both bassists played slightly laid-back in relation to the *pulse* and B2 used a larger delay (Pulse–B2 = 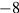 ms) than B1 (Pulse–B1 = 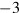 ms). Doing the same calculations for the HH and the CY onsets, Figure 6(b), shows that the CY is also played in a slightly laid-back fashion in relation to the *pulse*. In contrast, the HH (grey) is clearly played before the *pulse* in all recorded ensemble combinations.

However, comparing the asynchronies of the bassists to the pulse does not support the earlier assumption that the smallest intrapersonal asynchrony (here player D2) would result in the smallest interpersonal ensemble asynchrony. Figure 6(a) shows that B1 was almost perfectly synchronised with the pulse, when D2 was performing (Pulse–B1&D2 = 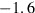 ms) but this was not the case for B2. B2 was closer to the pulse when D1 was playing (Pulse–B2&D1 = 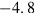 ms). Furthermore, both bassists played the most laid-back with D3 (Pulse–B1&D3 = 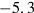 ms; Pulse–B2&D3 = 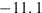 ms), who used smaller intrapersonal CY–HH asynchronies than D1.

To investigate the effect of the rhythm section players (bassists, drummers) on the asynchronies of BS, HH and CY with the *pulse* we conducted three separate two-way ANOVAS for each dependent variable. For the Pulse–BS asynchrony, we found a significant main effect of bassist [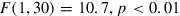] and drummer [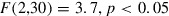]. In contrast, Pulse–CY asynchrony showed no significant main effects or interactions, whereas in the case of HH–pulse asynchrony a significant main effect of drummer [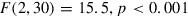] was found.

**Figure 7.**
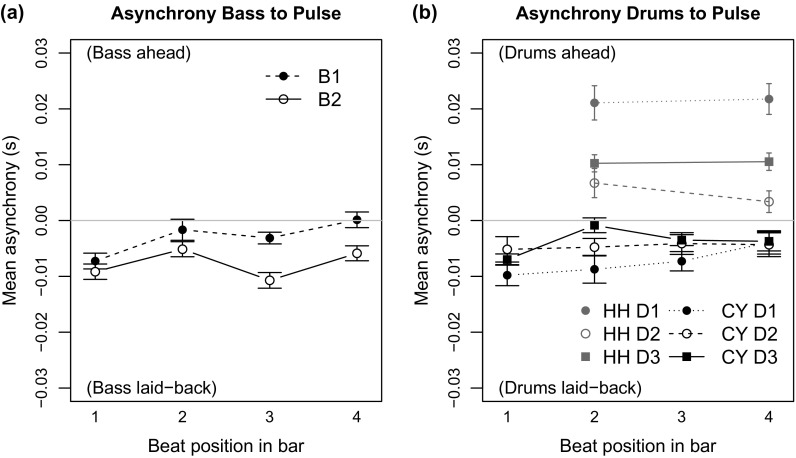
(a) Asynchronies between bass note onsets and the pulse were smaller for beats 2 and 4. (b) Asynchronies for hi-hat (grey) and cymbal taps (black) for all three drummers for each beat position in the bar. The error bars show the standard error of the mean.

A closer investigation of the asynchronies between the *pulse* and the rhythm section instruments for each quarter beat shows that the downbeats 1 and 3 of both bassists were systematically delayed, whereas the quarter notes of the ride cymbal showed more equal inter-beat intervals (Figure 7(a) and (b)). The hi-hat onsets on the backbeats were systematically played before the pulse, except in the case of D2, who placed the hi-hat closer to the *pulse*. The bassists’ delays, which were regular at the half-note level but not at the quarter note level, are in line with an observation made by Parsons and Cholakis (1995) and Butterfield (2006) who attributed such fluctuations to having the ability to create a sensation of ‘pushing the back-beats’ and to create a ‘sense of forward drive’.

## 3. Listening test

The listening test was designed to evaluate two hypotheses derived from the results of the production experiment. Taking into account that the measured ensemble asynchronies were up to 30 ms, but most ensemble combinations showed smaller asynchronies, we hypothesised that listeners today, who are accustomed to listen to computer produced beats, might prefer a fully quantised (dead-pan) rhythm section also for jazz performances. In more detail, we hypothesised that listeners would prefer perfectly synchronised performances when they listen to only the rhythm section (drums and bass) but may be more tolerant to ensemble asynchrony when a soloist plays on top of the beats.

### 3.1. Methods

#### 3.1.1. Participants

Participants in this study (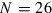, female = 13, male = 13) were between 20 and 36 years old (mean = 29.6). The group comprised professional musicians (15), professional dancers (4) as well as non-experts (7).

#### 3.1.2. Experimental design

From the recordings of the six ensembles in the production experiment, we selected two excerpts per ensemble, one for each song form (A and B) for the listening test. Each of the 12 excerpts had a duration of 15–20 s ^3^.

For each excerpt, we created 10 manipulated versions by altering the timing data of the drums and the bass. We took the timing deviations of the drum instrument onsets and the bass note onsets to the* pulse* and extrapolated (and reduced) these by 20, 40, 60, 80 and 100%, a procedure introduced by Kilchenmann and Senn (2015). The timing data of drums and bass was then re-synthesised in the R-statistics software, using samples of drum instruments (cymbal, hi-hat) and a synthetic bass sound. All 12 excerpts and their manipulations were created in two versions, one with only the sounds of the rhythm section and one containing also the original saxophone track.

A web-interface was programmed using the web audio API^4^ which allowed to load and start multiple sound files simultaneously. Loading the original version and the 10 manipulations, a slider on the screen can be moved to switch between the different timings on the fly while the music is playing. Aligning the audio files in the order from 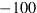 to +100%, would play the music with the original timing when the slider is in the middle. Moving the slider to the left would reduce the ensemble asynchronies, moving the slider to the right extrapolates the ensemble asynchronies.

To avoid having the midpoint of the slider always correspond to the original timing, only nine manipulations were presented to the participants (e.g. 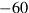 to +100%). To change the behaviour of the slider, the scale was inverted in some cases (e.g. +80 to 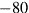
%). Four different listening orders were prepared to avoid effects of ordering. This resulted in a 6 (ensembles) 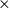 2 (songs) 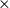 2 (rhythm section only/rhythm section with saxophone track) 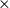 9 (asynchrony profiles) design for the experiment.

#### 3.1.3. Procedure

The listening test was provided online and participants were requested to use headphones and to work in a quiet environment. As a first step, the participants read the ethics approval information and provided consent. As the next step they entered background information (e.g. gender and age) and filled in a questionnaire about their musical background (e.g. whether they play a musical instrument, whether they are professional musicians or dancers). After a sound check, where the fundamental behaviour of the interface was introduced, the participants received instructions on the actual listening task. They listened to two extreme versions of the same jazz beat (+100 and 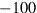
%) and were given a slider to select between the different timing versions, the same way as later in the experiment. The screen indicated: ‘Listen to the clip and adjust the slider to the position you prefer. You can listen as long as you want’. As the final introductory step, participants heard an example with the saxophone playing on top and had to move the slider again to change the timing of the drums and the bass. Below the slider they were given a four-point scale to choose how confident they were with their selection (Very confident—4, Confident—3, Not sure—2, I had to guess—1). The final instruction was: ‘Click on the Continue button to submit the slider position and your answer’. On the next page, participants were asked if they were ready for the experiment. After their confirmation, each participant was presented with the 24 sound examples (12 excerpts, with/without saxophone track), each individually with one slider and one confidence scale. The experiment lasted approximately 30 min, depending on how often the participants listened to each example.

### 3.2. Results

Overall, the participants preferred versions with asynchronies smaller than in the original recordings for all excerpts. In the data, we found no significant effects of gender, the expertise of the participants (e.g. musicians, dancers, non-musicians), and the listening order of presented stimuli. One participant stopped in the middle of the experiment and was omitted entirely from the analyses.

**Figure 8.**
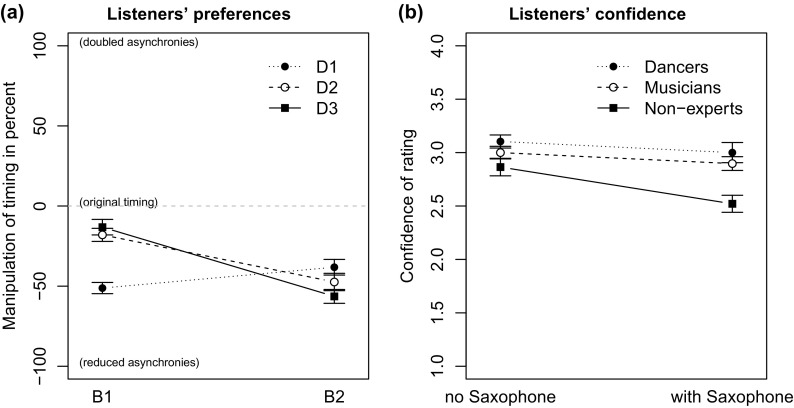
Results of listening experiment: (a) Preferred version of ensemble asynchrony manipulation grouped by the players. (b) Listeners’ confidence ratings grouped by their experience. The error bars are showing the standard error of the mean.

A four-way repeated measures ANOVA on the preferred version by properties of the original stimuli (bassist playing, drummer playing, song A/B, with/without saxophone track) was conducted. There were significant main effects of the bassist playing [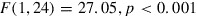] and the drummer playing [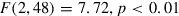], as well as a main effect for the presence of the saxophone track [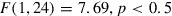] and an interaction between the bassist and the drummer [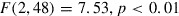]. Figure 8(a) shows the preferred versions of the stimuli grouped by bassists and drummers. Most important, the significant interaction between the bassist and the drummer [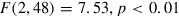] indicates that the listeners preference did primarily depend on the specific ensemble combination. No main effect of the performed song, or any other significant interaction was found.

For stimuli that involved B1, a version significantly closer to the original timing (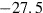
%) was preferred by the listeners than for B2 (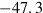
%). A reason for these differences can be explained by the properties of the original recordings. Looking at the asynchronies measured in the playing experiment, Figure 6(a) shows that B1 originally played closer to the *pulse* (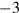 ms) than B2 (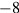 ms) and listeners preferred versions with bass asynchronies smaller than 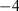 ms to the *pulse* (B1 = 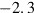 ms and B2 = 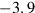 ms).

Also for the drummers, we found that for D2 (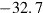
%) and D3 (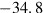
%) participants preferred less manipulated (reduced asynchrony) versions than for the drummer D1 (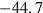
%). Looking at the asynchronies of the drummers in Figure 6(b) one can see that the HH of D2 and D3 was played closer to the *pulse*. The original mean asynchronies to the pulse were in a range of up to +25 ms (early HH of D1 playing with B2), whereas the preferred version had reduced asynchronies 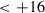 ms for the HH and 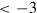 ms for the bass. This would result in overall discrepancies smaller than 19 ms between bass and drum sounds. Other studies also reported that PDs up to 20 ms were mostly irrelevant to the perception of groove level, while PDs larger than 30 ms showed a negative effect on perceived groove (Butterfield, 2010; Kilchenmann & Senn, 2015). Our findings support that listeners have a preference for asynchronies smaller than 20 ms for jazz grooves; however, we did not find support for our earlier hypothesis that a fully synchronised version would be preferred.

The significant main effect of the presence of the saxophone track showed that listeners preferred a version with larger asynchronies when listening to stimuli with the saxophone than when listening to stimuli with only the rhythm section. This finding is in line with our second hypothesis that with a soloist, listeners are more tolerant to ensemble asynchronies.

A mixed model two-way ANOVA on the confidence ratings by saxophone track (within) and listeners expertise as the between factor showed that listeners were more confident with their ratings when no soloist was playing [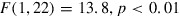], independent of their expertise (see Figure 8(b)). A possible explanation may be that the listening focus changes from the rhythm section to the melody and the sound of the soloist when the saxophone track was added.

A limitation in this experiment was that we did not manipulate the timing of the saxophone track. Consequently, it could also be possible that listeners preferred a less manipulated version of the rhythm section with the saxophone track because the timing of the saxophonist fits better with the original recording. However, participants still preferred a version with reduced asynchronies in the rhythm section.

## 4. General discussion

The purpose of this study was to investigate the temporal and dynamical structure of jazz performance as influenced by the varied personnel in the rhythm section. The results provided five main findings that contribute to the literature. First, all combinations of performers played with faster tempi than introduced, which was directly influenced by the drummer. Second, all performers demonstrated a higher precision of regularity at the tactus level of the beats 2 and 4 (backbeat) than for the quarter beats 1 and 3. Third, timing effects related to drummers’ microstructural asychronies were found between the hi-hat and the ride cymbal. The observation of the hi-hat playing before the pulse of the music, especially supports Butterfield’s theory that an early hi-hat is used by jazz drummers to create an effect of anacrusis, that induces a powerful sense of forward drive and therefore contributes to a certain quality of push in jazz grooves (Butterfield, 2006). Fourth, interpersonal timing relationships were dominantly controlled by the drummers’ hi-hat use. This indicates that the hi-hat plays a key role in jazz ensemble timing. Fifth, swing ratios and note velocities were performer-dependent but also influenced by the tempo, which is in line with findings of Benadon (2009) and Friberg and Sundström (2002). All these observations together show how the combination of different musicians in a jazz ensemble contributes to variations in the timing and accentuation profiles of each ensemble combination and creates unique interpretations of jazz songs.

Early studies on synchronisation in jazz performance primarily focused on the synchronisation between bass note onsets and ride cymbal taps (Keil, 1987; Prögler, 1995). The conclusion drawn from these measurements was that observed asynchronies (*Participatory Discrepancies*, PDs) between these two instruments induce an element of *groove* in the performance. A possible reason why bass notes and cymbal taps were chosen as the reference instruments was that these were easy to extract from mixed audio recordings because of their non-overlapping frequency bands. However, systematic reproductions of such PDs failed (Butterfield, 2010; Davies, Madison, Silva, & Gouyon, 2013). It is only recent that the intrapersonal timing of the drummers has received more attention (Frühauf et al., 2013). When Kilchenmann and Senn (2015) studied a duo consisting of a drummer and a bassist, they observed that the drummer’s hi-hat was played significantly earlier than the rest of the instruments. A leading hi-hat was also mentioned by Butterfield (2010). Our measurements underline these observations. Furthermore, by comparing the three different professional jazz drummers in this study, we found that each drummer showed a personal hi-hat timing profile that affected the synchronisation in the rhythm section. This strengthens our assumption that the hi-hat on the *backbeats* might play a key role in the synchronisation of the jazz rhythm section and deserves more attention in future performance research.

With the listening test, we were able to show that listeners preferred versions of the stimuli with at least as accurate synchronisation as in the original recordings or better. For ensemble players who already played more *tight*, the listeners showed a tendency to prefer a version closer to the original timing. Fully quantised performances were not selected as the preference for a jazz rhythm section, an observation similar to that in Davies et al. (2013), Kilchenmann and Senn (2015), and Senn et al. (2016). All studies have in common that fully quantised jazz beats were rated as less groovy or less likely to induce body movements.

The largest hi-hat asynchronies measured in the recorded performances were around 26 ms ahead of the pulse, but listeners showed a preference for ensemble asynchronies of less than 19 ms, clearly smaller. This finding may be explained by temporal masking of sounds falling into small time windows. Depending on the rise time of the tones and the dynamics, masking effects lead to the perception of only one event in such a case (Rasch, 1978). This perceptual property of human hearing may define a threshold for a *tight-interlocked rhythm section* to produce asynchronies smaller than 20 ms, which corresponds to perceptual results reported in the literature (for a comprehensive discussion, see Goebl, 2003, p. 89ff.).

A limitation in this study might be that we quantised all physical onsets from different instruments the same way. Perceptual experiments have shown that different sounds (e.g. double bass vs. drums) with different attack times can influence the perceived timing (Gordon, 1987; Vos & Rasch, 1981). As a consequence physically quantised bass and drum onsets might not have been perceived as dead-pan. However, in our case the hi-hat, with a shorter attack time than a bass tone, was originally played earlier than the bass. In this way, the applied quantisation must have reduced the asynchrony between both instruments.

Another reason for the preference of a *tight* but not *dead-pan* rhythm section might be that the vertical quantisation also alternates the timing on the horizontal level, when all onsets are aligned to the mean pulse. This leads to an unnatural high timing precision with a machine like aesthetic, untypical for jazz music, but characteristic for EDM. Janata et al. (2012, p. 71) asked if ‘high-groove music essentially serves as an invitation to join the group [..] to what extent are [..] timing deviations from metronomic timing [..] indicative of social interaction, in the sense that they help differentiate human and computer time keepers?’

In this study, we transformed the interactive timing of the jazz trios into *dead-pan* computer timing and found that such extreme manipulations were not endorsed by the listeners. So-called humaniser functions in audio software, can add timing variability to a *dead-pan* beat. These functions can either generate random numbers or follow a systematic pattern^5^. An interesting aspect to investigate in the future is how listeners perceive human timing variability in contrast to computer added timing variability based on such algorithms. Apart from the properties of the music itself, there may also be contextual influences on how listeners perceive groove based music. These influences can range from personal preferences based on the musical taste, a listener’s mood (tired, sad, or exhausted vs. happy or energetic), to the environment where they listen to the music (at home, in a club venue at high volume levels, or in a laboratory with headphones). While one might enjoy a band live on stage and dance with friends to the music, listening to the same music at home may not trigger the same excitement and urge to move. In the future, it would be interesting to investigate such contextual aspects of groove based music from both perspectives, the listeners’ and the performers’.

## Footnotes

1 For example listen to ‘The Cannonball Adderley Quintet—At The Lighthouse’, Riverside Records (RLP 9344, 1960).

2 Note events are saved as double precision time stamps in the Ableton-Live Project File and were directly extracted from the XML-format-based Ableton project file using the ’XML’ package (ver. 3.98) in R-statistics.

3 For the listening experiment, only the onsets of the ride cymbal (CY), the hi-hat and the bass note onsets were used.

4
https://www.w3.org/TR/webaudio/.

5 For example, see the ‘Group Harmonizer’ plugin by James Holden available for Ableton Live, based to an algorithm presented in Hennig (2014)
